# High genetic predisposition and vertebral fracture burden in pregnancy and lactation-associated osteoporosis: clinical insights from a Taiwanese cohort

**DOI:** 10.3389/fendo.2026.1930247

**Published:** 2026-10-06

**Authors:** Wei-Yih Chiu, Yu-An Chen, Shyang-Rong Shih, Rong-Sen Yang, Ming-Hsiao Hu, Wen-Ping Yang, Pei-Lung Chen, Keh-Sung Tsai, Wei-Shiung Yang

**Affiliations:** 1 Division of Endocrinology and Metabolism, Department of Internal Medicine, National Taiwan University Hospital and National Taiwan University College of Medicine, Taipei, Taiwan; 2 Department of Laboratory Medicine, National Taiwan University Hospital, Taipei, Taiwan; 3 Graduate Institute of Medical Genomics and Proteomics, College of Medicine, National Taiwan University College of Medicine, Taipei, Taiwan; 4 Department of Medical Research, National Taiwan University Hospital Hsin-Chu Branch, Hsinchu, Taiwan; 5 Department of Orthopedic Surgery, National Taiwan University Hospital, Taipei, Taiwan; 6 Department of Metabolism and Endocrinology, Taipei City Hospital, Renai Branch, Taipei, Taiwan; 7 Department of Medical Genetics, National Taiwan University Hospital, Taipei, Taiwan; 8 Graduate Institute of Clinical Medicine, College of Medicine, National Taiwan University, Taipei, Taiwan

**Keywords:** bone mineral density (BMD), genetic predisposition, postpartum back pain, pregnancy and lactation-associated osteoporosis (PLO), vertebral fragility fractures, whole-exome sequencing (WES)

## Abstract

**Background:**

Pregnancy and lactation-associated osteoporosis (PLO) is a rare disorder causing rapid bone loss and severe fragility fractures. While familial clusterings suggest genetic susceptibility, the specific clinical features, genetic landscape, and skeletal recovery trajectory of PLO remain poorly characterized in non-Caucasian populations. This study aimed to evaluate a Taiwanese PLO cohort to optimize diagnostic awareness.

**Methods:**

Eleven postpartum women with PLO were evaluated using bone densitometry and spinal imaging. Whole-exome sequencing was performed utilizing a targeted 398-gene skeletal disease virtual panel. Patients were followed longitudinally for a median of 25 months during lactation cessation and/or targeted bone therapy.

**Results:**

Seven women developed symptoms after their first pregnancy and four after their second. Vertebral fractures occurred in 91% (10/11) of patients, 80% (8/10) of whom had multi-level involvement predominantly localized to the thoracolumbar spine (T3-L5, peaking at T11-L2). Baseline lumbar bone mineral density (BMD) was significantly lower than hip sites (*P* = 0.018). Genetic variants were identified in 10 of 11 patients (91%), with six individuals harboring pathogenic variants in *WNT1*, *LRP5*, *COL10A1*, *LTBP3*, and *CALCR*. Patients with pathogenic variants exhibited significantly lower lumbar spine BMD than those without (Z-scores: -3.7 vs. -3.0; *P* = 0.043). Post-treatment, the cohort’s median Z-score improved from -3.5 to -2.3 (*P* = 0.005), though none achieved a score above -1.0.

**Conclusions:**

Our findings suggest a significant genetic burden associated with PLO, potentially revealed by pregnancy and lactation stressors. The predominance of vertebral bone loss highlights acute postpartum back pain as a notable clinical sign warranting further investigation. Optimizing long-term maternal outcomes may require rapid diagnostic evaluation, prompt lactation cessation, and the future integration of genotype-informed management strategies.

## Introduction

Pregnancy and lactation-associated osteoporosis (PLO) is a rare skeletal disorder characterized by early-onset osteoporosis, often manifesting as vertebral compression fractures or transient bone loss of the hip during late pregnancy or breastfeeding (1–4). Epidemiological data on the incidence of PLO are limited, but estimates vary, including 6.8 cases per 100,000 pregnancies in the UK (Edinburgh) (5) and 460 cases per million deliveries in Japan (6). The exact causes of PLO remain unclear, and the condition is often associated with significant morbidity. It is believed that PLO is related to hormonal changes during pregnancy and lactation. Nevertheless, PLO is extremely rare in normal pregnancies, implying that additional factors may contribute to its development. Peris et al. (7) reported that 53% of first-degree relatives of PLO patients had osteoporosis, compared to 15% in matched controls, with multiple affected members within the same families, suggesting an underlying genetic predisposition to low peak bone mass. A German study identified risk variants in bone-related genes such as *LRP5, WNT1* and *COL1A1/COL1A2* in nearly 50% of PLO cases (8), and a recent exome-sequencing study of 110 North American women with PLO reported rare predicted deleterious variants in 30%, most frequently in WNT-pathway genes (*LRP5*, *LRP6* and *WNT1*) (9). The broader clinical phenotype, genetic architecture, and longitudinal skeletal recovery remain poorly characterized in Asian populations. This data gap often results in diagnostic delays and missed interventions for women presenting with debilitating postpartum back pain. This study aimed to characterize the clinical features and skeletal recovery of a Taiwanese PLO cohort, utilizing whole-exome sequencing (WES) to identify underlying genetic susceptibilities and improve diagnostic awareness for this severe maternal condition.

## Methods

### Participants and ethical approval

PLO is clinically characterized by vertebral compression fractures or significant hip bone loss during pregnancy or within 6 months postpartum (1-3). We retrospectively enrolled all women diagnosed with PLO between 2017 and 2026 at our institution’s Endocrinology Department. Prior to diagnosis, secondary causes of osteoporosis were excluded, including celiac disease, idiopathic hypercalciuria, chronic kidney disease (estimated glomerular filtration rate less than 30 ml/min), primary hyperparathyroidism, and chronic corticosteroid use. This study was conducted in accordance with the Declaration of Helsinki and received approval from our hospital’s Research Ethics Committee (reference number: 201508107RIND). All participants provided written informed consent for the evaluation of osteoporosis and its underlying genetic basis after a comprehensive explanation of the study objectives and procedures.

### Radiological assessment

Bone mineral density (BMD) of the hip and lumbar spine was assessed using dual-energy X-ray absorptiometry (DXA). In accordance with international guidelines (10) for premenopausal women, Z-scores were utilized instead of T-scores to ensure an appropriate age-matched reference for evaluating bone mass in younger populations. X-ray and magnetic resonance imaging (MRI) were employed to identify the locations of fractures, with a primary focus on the thoracic and lumbar spine, as well as areas of reported bone pain. Vertebral fractures on X-ray were evaluated using the semi-quantitative method proposed by Genant et al. (11). DXA scans and X-rays were obtained at baseline and during follow-up, with MRI reserved for patients presenting with multiple fractures or when the underlying condition could not be determined.

### Whole-exome sequencing and bioinformatics analysis

Genomic DNA from all patients was subjected to WES. Exonic regions were enriched using the Agilent SureSelect Human All Exon V6 Kit following the manufacturer’s protocol. Sequencing was carried out on the Illumina NovaSeq 6000 platform to generate 150 bp paired-end reads. Raw sequencing data were processed using the Illumina DRAGEN Bio-IT Platform (v4.2; Illumina Inc., San Diego, CA, USA). Reads were aligned to the human reference genome (GRCh38), and variant calling was performed according to the DRAGEN (12) pipeline best practices. Annotation of genetic variants was carried out using the Ensembl Variant Effect Predictor (VEP v.111) (13).

A custom-designed gene panel consisting of 398 genes associated with bone mass regulation, skeletal dysplasias, dysostoses, and connective tissue disorders, as listed in **Supplementary Table 1**, was applied for focused analysis. The complete filtering and interpretation workflow is summarized in **Supplementary Figure 1**. Variants were retained for prioritization only if they carried a “PASS” filter status, a minor allele frequency (MAF) of 0.01 or less in the Genome Aggregation Database (gnomAD), a read depth (DP) of at least 5, and a variant allele fraction (VAF) of at least 20%. Retained variants were then triaged into three classes: (i) loss-of-function variants, comprising frameshift, stop-gain, and canonical splice-site variants; (ii) nonsynonymous variants, comprising missense, in-frame indel, stop-loss, and initiation-codon variants, predicted to be protein-damaging by Combined Annotation Dependent Depletion (CADD) (14) at a phred score of 25 or above, or by Deleterious Annotation of genetic variants using Neural Networks (DANN) at a score of 0.93 or above where a CADD score was unavailable; and (iii) splicing-region variants with a predicted splicing impact (SpliceAI (15) delta score of 0.5 or above). The resulting candidate set was restricted to the 398-gene virtual panel and was screened in parallel for variants or genes recurring across samples. Panel-restricted candidates were classified according to the five-tier system recommended by the American College of Medical Genetics and Genomics/Association for Molecular Pathology (ACMG/AMP) guidelines (16) by manual review, supported by an in-house developed workflow, the Gendiseak (GDK) platform (17, 18), and cross-checked against ClinVar (January 2025 release) (19), VarSome (June 2025) (20), and GeneBe (June 2025) (21). Allele frequencies in the gnomAD East Asian subset and in the Taiwan Biobank (TWB) were additionally retrieved and are reported for all disease-causing variants.

In parallel with the prioritization pathway described above, and independently of the allele frequency filter, variants already reported as pathogenic or likely pathogenic in ClinVar, confirmed in MITOMAP, or pathogenic/likely pathogenic in a gene on the ACMG secondary findings list were also reviewed (**Supplementary Figure 1**, pathway 1). This step was performed as a supplementary check of the annotation pipeline and did not form part of the primary analysis; no variant relevant to PLO and no reportable secondary finding was identified through it, and its output is therefore not included in the variant counts reported in the Results.

For downstream analyses, variants were categorized as follows. Disease-causing variants (DCVs) were defined as heterozygous variants classified as pathogenic or likely pathogenic (P/LP) by manual review, VarSome, or GeneBe, in genes with autosomal dominant (AD) or combined autosomal dominant/autosomal recessive (AD/AR) inheritance. Relevant variants (RVs) were a collection of genetic variants potentially associated with bone fragility and skeletal disorders, including DCVs, heterozygous P/LP variants in AR inheritance pattern, as well as variants of uncertain significance (VUS) regardless of inheritance mode. Gene-disease inheritance information was obtained from the Gene Curation Coalition (GenCC) database.

### Statistical methods

Continuous variables were presented as median (Q1, Q3), whereas categorical variables were expressed as frequencies (percentages). The rates of all outcomes were calculated with 95% confidence intervals (CIs). BMD Z-scores were compared across anatomical regions, as well as at two time points (diagnosis and 2–3 years post-treatment), using the Wilcoxon signed-rank test. To compare subgroup differences based on genetic variants, the Wilcoxon rank-sum test for continuous data and the Fisher’s exact test for categorical data were used. A *P*-value less than or equal to 0.05 was considered statistically significant. All statistical analyses were conducted using Stata (version 12.1; StataCorp, College Station, TX, USA).

## Results

### Clinical and radiographic features of PLO

The study cohort comprised 11 women with clinically diagnosed PLO, with a median age of 35 years (34, 37) (**Table 1**). These patients had the following demographic characteristics: median height of 160 cm (155, 165), weight of 51 kg (43, 63), and body mass index of 21.0 kg/m² (17.9, 24.6). All patients developed symptoms during lactation, with onset after the first delivery in 7 cases and after the second delivery in 4 cases. Symptom occurred at a median of 3 months postpartum (1, 4). Seven women presented within the first 3 months, and four between 4 and 6 months. Six of the patients experienced height loss, with a median height loss of 4.5 cm among these patients (3, 9), while the median height reduction for all 11 patients was 3.0 cm (0, 5). Back pain was the predominant presentation in all patients (11 cases).

**Table 1:** Clinical and radiographic characteristics of PLO patients with and without disease-causing variants (DCVs).

Characteristics	Total (n = 11)	Without DCVs (n = 5)	With DCVs (n = 6)	*P*-value^*^
Age at diagnosis, years	35 (34, 37)	37 (35, 38)	34 (30, 36)	0.054
Height, cm	160 (155, 165)	159 (155, 163)	161 (156, 165)	0.715
Weight, kg	51 (43, 63)	49 (46, 65)	53 (42, 58)	0.584
Body mass index, kg/m^2^	21.0 (17.9, 24.6)	22.5 (18.4, 25.7)	19.7 (17.1, 23.8)	0.361
Height decrease, cm	3 (0, 5)	0 (0, 4)	3 (0, 5)	0.630
Parity				
1	7 (63.6%)	2 (40%)	5 (83%)	0.242
2	4 (36.4%)	3 (60%)	1 (17%)	0.242
Disease onset during lactation				
In the first 3 months after childbirth	7 (63.6%)	3 (60%)	4 (67%)	1.000
In the 4–6 month postpartum period	4 (36.4%)	2 (40%)	2 (33%)	1.000
Interval between delivery and disease onset, months	3 (1, 4)	3 (1, 4)	2.5 (1, 4)	0.925
Breastfeeding, months	6 (4, 8)	6 (4, 8)	5.5 (4, 6)	0.782
Lumbar spine BMD, Z-score	-3.5 (-3.8, -3.0)	-3.0 (-3.3, -2.5)	-3.7 (-4.1, -3.5)	**0.043**
Femoral neck BMD, Z-score	-1.3 (-2.0, -1.1)	-1.1 (-2.0, -0.4)	-1.65 (-2.25, -1.2)	0.280
Total hip BMD, Z-score	-1.6 (-2.4, -0.7)	-0.7 (-3.1, 0.1)	-1.95 (-2.35, -1.2)	0.480
Vertebral fractures	10 (91%)	4 (80%)	6 (100%)	0.455
Vertebral fractures, multiple (at least 2 vertebrae)	8 (73%)	2 (40%)	6 (100%)	0.061
Vertebral fracture number per case	4 (1, 8)	1 (1, 7)	4 (3, 8)	0.312

Continuous variables are presented as median (Q1, Q3), and categorical variables are expressed in frequency (percentage).

^*^P-values were calculated to compare characteristics between patients with and without DCVs, using the Wilcoxon rank-sum test for continuous variables and Fisher’s exact test for categorical variables.

Bold values indicate *P* < 0.05.

At diagnosis, the median BMD Z-scores were significantly reduced: -3.5 (-3.8, -3.0) at the lumbar spine, -1.3 (-2.0, -1.1) at the femoral neck, and -1.6 (-2.4, -0.7) at the total hip. Furthermore, the lumbar spine demonstrated a significantly lower BMD compared to the femoral neck (-3.5 vs. -1.3, *P* = 0.018) and the total hip (-3.5 vs. -1.6, *P* = 0.018) region of interest, with statistically significant differences confirmed by Wilcoxon signed-rank tests.

Among the 11 PLO women, nine experienced only vertebral compression fractures. One patient had a combination of vertebral and left pubic bone fractures. The remaining patient had fractures at the left sacral ala and bilateral pubic bones. Among the ten patients with vertebral fractures, a total of 58 fractured vertebrae were identified, yielding a median of 4 fractures per patient (1, 8). These spinal fractures occurred between T3 and L5, particularly at T11–L2 (**Figure 1**). Eight patients sustained multiple spinal fractures (≥2 sites), and the most severe case involved 14 levels. Review of pre-pregnancy medical histories revealed no prior fractures in any of the patients.

**Figure 1:**
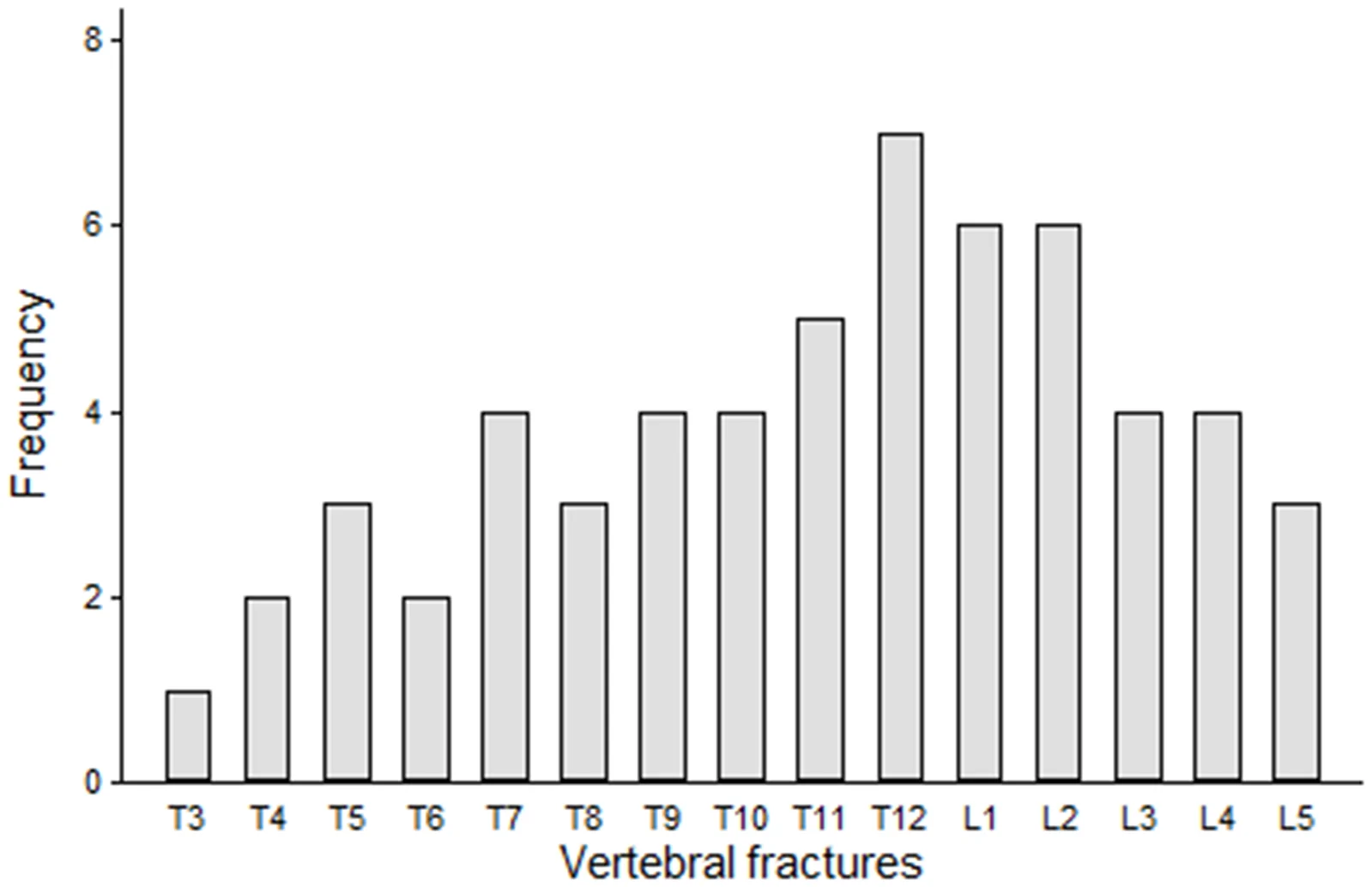
Distribution of Fractured Vertebral Levels in PLO. Ten out of 11 PLO women had vertebral compression fractures, affecting a total of 58 vertebrae. These fractures predominantly occurred in the thoracolumbar region, specifically between T3 and L5, with a high frequency observed at the levels of T11 to L2.

### Genetic analysis results: 91% of PLO patients had relevant variants, and 55% carried disease-causing variants

Whole-exome sequencing using a customized 398-gene skeletal disease virtual panel was performed on 11 women with PLO, identifying heterozygous variants in 10 cases (**Figure 2**). Based on the prior PLO study’s classification framework (8), relevant variants (RVs) were defined as genetic variants in skeletal disorder-related genes meeting ACMG/AMP categories II–V criteria. Our analysis revealed that 91% of PLO patients carried RVs (**Table 2**). Disease-causing variants (DCVs), a subset of RVs classified as ACMG/AMP categories IV and V, were identified in six PLO patients (55%). Seven DCVs were detected in these six women, affecting five genes: *WNT1* (n = 2), *LRP5* (n = 2), *LTBP3* (n = 1), *COL10A1* (n = 1) and *CALCR* (n = 1); one woman (Case 8) carried two DCVs, one in *COL10A1* and one in *LRP5*, and is counted once in the cohort-level proportion (**Table 3**). All identified DCVs were visually confirmed by manual review of read alignments in the Integrative Genomics Viewer.

**Figure 2:**
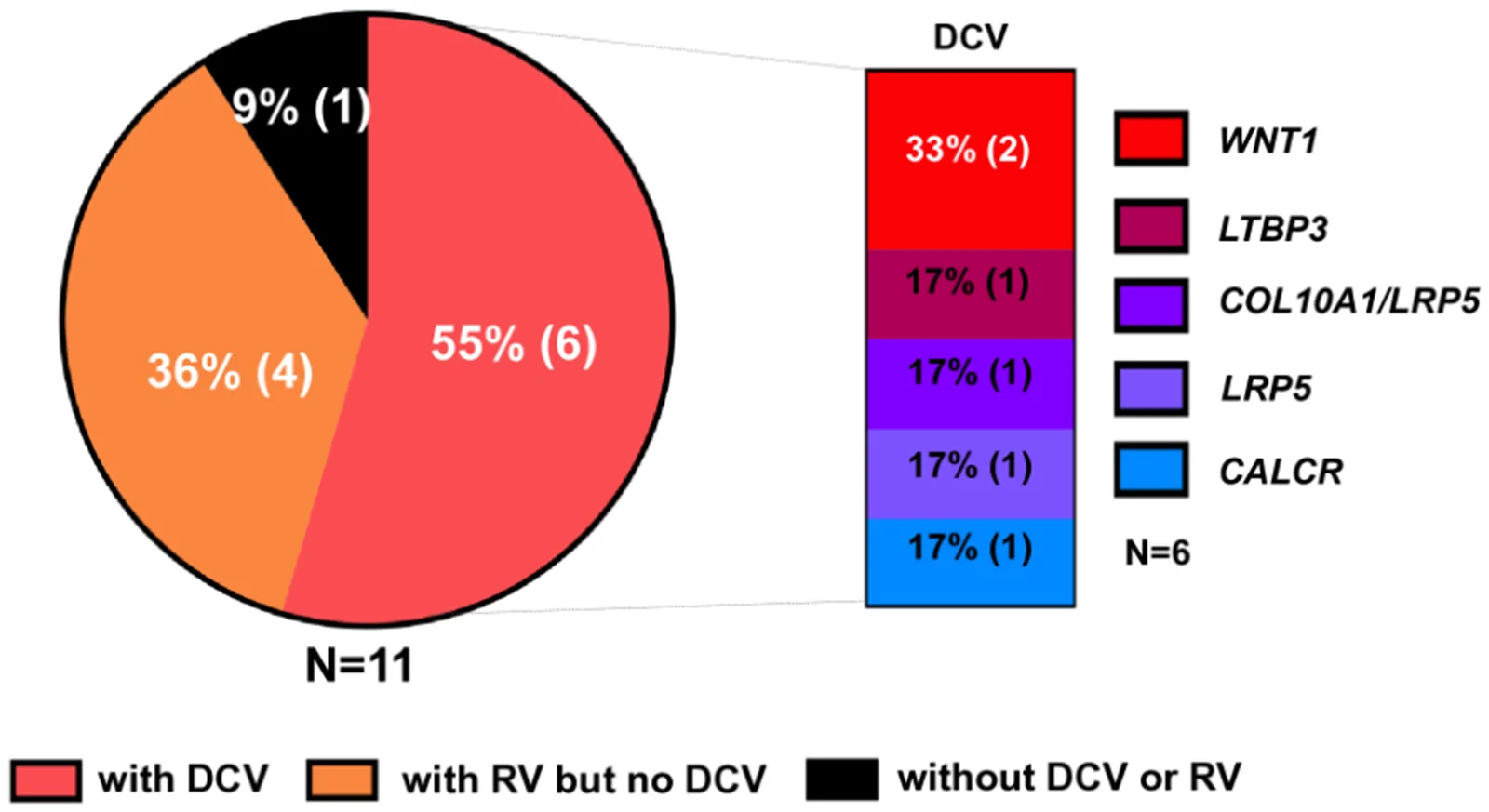
Genetic landscape and cohort distribution based on variant status. The pie chart classifies the 11 women by variant profile: those with at least one disease-causing variant (DCV; with or without concurrent relevant variants [RVs]), those with RVs but no DCV, and those with neither. The adjacent panel lists the genes affected by the seven DCVs identified in the six DCV-positive women. The “*COL10A1*/*LRP5*” entry denotes the single woman (Case 8) who carried two DCVs, one in each of these genes; she is counted once in the pie chart. Gene-level counts in this panel correspond to the DCV rows of **Table 2** and to **Table 3**.

**Table 2:** Case-level summary of disease-causing variants (DCVs) and relevant variants (RVs) identified by virtual panel analysis in the PLO cohort.

Case	Category	Gene	HGVSc	HGVSp	ACMG classes	VARSOME (2025 June)	Genebe (2025 June)	ClinVar (2025_Jan)	MOI (GenCC)
1	DCV	*WNT1*	NM_005430.4:​c.677C>T​	​p.(Ser226Leu)	LP	LP	LP	Not reported	AD/AR
	RV	*IDUA*	NM_000203.5:​c.1597C>T​	p.(Pro533Ser)	LP	LP	VUS	P(1) VUS(1)	AR
	RV	*FGFR2*	NM_000141.5:​c.253A>G​	p.(Thr85Ala)	VUS	VUS	VUS	Not reported	AD
	RV	*P3H1*	NM_022356.4:​c.326T>G​	​p.(Phe109Cys)	VUS	VUS	VUS	VUS (1)	AD/AR
2	RV	*COL27A1*	NM_032888.4:​c.3786T>A​	p.(Tyr1262*)	P	LP	P	Not reported	AR
	RV	*MCM5*	NM_006739.4:​c.1976-9T>A		VUS	VUS	VUS	Not reported	Unknown
3	RV	*PIK3C2A*	NM_002645.4:​c.1352T>C​	p.(Ile451Thr)	VUS	VUS	VUS	VUS (1)	AR
	DCV	*WNT1*	NM_005430.4:​c.291C>A​	p.(Phe97Leu)	VUS	LP	VUS	Not reported	AD/AR
4	RV	*FAM20C*	NM_020223.4:c.956 + 12_956 + 13insGTCCTCCCTCCATCCGCGACAGGTGAGCCCTTCC		VUS	VUS	VUS	Not reported	AR
5	not found								
6	DCV	*LTBP3*	NM_001130144.3:​c.1936G>A​	p.(Gly646Ser)	VUS	VUS	LP	Not reported	AD/AR
	RV	*PAPSS2*	NM_001015880.2:​c.397C>T	p.(Arg133Cys)	VUS	VUS	VUS	VUS (1)	AR
7	RV	*COL5A2*	NM_000393.5:​c.4352C>T	p.(Ser1451Phe)	VUS	VUS	VUS	Not reported	AD
	RV	*EVC*	NM_153717.3:​c.769C>A	p.(Leu257Ile)	VUS	VUS	VUS	Not reported	AD/AR
	RV	*VPS33A*	NM_022916.6:​c.1302 + 5G>A		VUS	VUS	VUS	Not reported	AR
8	DCV	*COL10A1*	NM_000493.4:​c.1228G>C​	​p.(Gly410Arg)	VUS	VUS	LP	Not reported	AD
	RV	*GHSR*	NM_198407.2:​c.107_109delAGC​	​p.(Gln36del)	VUS	VUS	VUS	Not reported	AD/AR
	DCV	*LRP5*	NM_002335.4:​c.3608C>T​	​p.(Ala1203Val)	VUS	VUS	LP	Not reported	AD/AR
9	RV	*ACAN*	NM_001369268.1:​c.7369A>G​	p.(Met2457Val)	VUS	VUS	VUS	Not reported	AD/AR
	DCV	*CALCR*	NM_001164737.3:​c.520A>T	​p.(Arg174*)	VUS	LP	VUS	Not reported	Unknown
	RV	*CFAP410*	NM_004928.3:​c.674G>C​	p.(Arg225Pro)	VUS	VUS	VUS	Not reported	AD
	RV	*MEOX1*	NM_004527.4:​c.593G>A​	p.(Arg198Gln)	VUS	VUS	VUS	VUS (1)	AD/AR
10	RV	*CRTAP*	NM_006371.5:​c.143A>T​	​p.(Tyr48Phe)	VUS	VUS	VUS	VUS (2)	AD/AR
	RV	*CWC27*	NM_005869.4:​c.427C>T​	p.(Arg143*)	P	P	P	P(3)	AR
	RV	*DSPP*	NM_014208.3:​c.2024_2025insGGACAGCAGCAGTAGCAG​	p.(Ser674_Ser675insArgAspSerSerSerSer)	VUS	VUS	VUS	Not reported	AD
11	DCV	*LRP5*	NM_002335.4:c.350G>A	p.(Trp117*)	P	LP	P	Not reported	AD
	RV	*ASCC1*	NM_001198800.3:c.317dup	​p.(Gly107TrpfsTer15)	P	LP	P	Not reported	AR
	RV	*FKBP10*	NM_021939.4:c.745C>T	p.(Gln249*)	P	LP	P	Not reported	AR
	RV	*LRRK1*	NM_024652.6:c.3439G>T	p.(Ala1147Ser)	VUS	VUS	LP	VUS (1)	AR

All variants were heterozygous. Annotations included manual ACMG classes, VarSome (June 2025), GeneBe (June 2025), ClinVar (Jan 2025), and mode of inheritance (MOI; GenCC). Abbreviations: AD, autosomal dominant; AR, autosomal recessive; HGVS, Human Genome Variation Society nomenclature (coding level); HGVSp, HGVS description at the protein level; LP, likely pathogenic; MOI, mode of inheritance; P, pathogenic; VUS, variant of uncertain significance. Case 5 carried no variant meeting the RV criteria. Case 8 carried two DCVs, one in *COL10A1* and one in *LRP5*; the seven DCVs listed here were therefore identified in six women, and Case 8 is counted once in the cohort-level DCV proportion of 6/11 (55%). This is the basis for the “*COL10A1*/*LRP5*” category shown in **Figure 2**. Note: for variant NM_001164737.3 (*CALCR*):c.520A>T the variant allele fraction (VAF) was 0.29.

**Table 3:** The seven disease-causing variants (DCVs) identified by virtual panel analysis in six of the 11 women with PLO (55%).

Gene	HGVSc	Protein level	ACMG classes	AF in gnomAD v4.1.0	AF in gnomAD v4.1.0 East-Asian	AF in TWB	ClinVar (2025_Jan)	CADD	SIFT	Polyphen-2	Bayes Del_addAF
*WNT1*	NM_005430.4:c.677C>T​	​p.(Ser226Leu)	LP	0.000001240	0.00004468	0	Not reported	30	Damaging	Damaging	Damaging
*WNT1*	​NM_005430.4:​c.291C>A​	p.(Phe97Leu)	LP	0.000004960	0.0001783	0.0005	Not reported	27	Damaging	Damaging	Damaging
*LTBP3*	NM_001130144.3:​c.1936G>A	p.(Gly646Ser)	LP	Not found	Not found	0	Not reported	30	Damaging	Damaging	Damaging
COL10A1^*^	NM_000493.4:​c.1228G>C​	​p.(Gly410Arg)	LP	0.000001239	0.00004458	0	Not reported	25	Damaging	Damaging	Damaging
LRP5^*^	NM_002335.4:​c.3608C>T	​p.(Ala1203Val)	LP	0.000	0.000	0	Not reported	21	Damaging	Damaging	Damaging
*LRP5*	NM_002335.4:c.350G>A	p.(Trp117*)	P	0.000	0.000	0	Not reported	40	n/a	n/a	Damaging
*CALCR*	NM_001164737.3:​c.520A>T​	​p.(Arg174*)	LP	Not found	Not found	0	Not reported	29	Damaging	n/a	Tolerant

Annotations include manual ACMG classes, ClinVar (Jan 2025) and in silico predictors. Allele frequencies (AF) were obtained from gnomAD v4.1.0, its East Asian subset, and the Taiwan Biobank (TWB). Variants marked with an asterisk (*) co-occurred in the same patient (Case 8). All variants were heterozygous. In silico predictions were used only as supporting evidence within the ACMG/AMP framework; none of these variants underwent functional or segregation validation. Abbreviations: BayesDel_addAF, BayesDel with allele frequency; CADD, Combined Annotation Dependent Depletion; LP, likely pathogenic; P, pathogenic; PolyPhen-2, Polymorphism Phenotyping v2; SIFT, Sorting Intolerant From Tolerant; n/a, not applicable. Note: for variant NM_001164737.3 (*CALCR*):c.520A>T the variant allele fraction (VAF) was 0.29.

### Subgroup differences based on the genetic variants

To evaluate the impact of genetic status on clinical presentation of PLO, we compared clinical and radiographic features between patients with and without DCVs (**Table 1**). No significant differences were found in anthropometrics, vertebral fracture occurrence, or hip BMD Z-scores. In contrast, lumbar spine BMD Z-scores were significantly lower in patients with DCVs compared to those without (-3.7 vs. -3.0, *P* = 0.043, Wilcoxon rank-sum test). Statistical analysis revealed a trend toward significance for a younger age at diagnosis (34 vs. 37, *P* = 0.054) and a higher proportion of multiple vertebral fractures (100% vs. 40%, *P* = 0.061) in patients with DCVs (**Table 1**).

### Treatments and follow-up

The lumbar spine BMD Z-scores at diagnosis and after a median follow-up of 25 months (23.5, 31) are illustrated in **Figure 3**. Upon PLO diagnosis, all 11 patients discontinued breastfeeding and initiated standard combined supplementation consisting of calcium (600–800 mg/day) and vitamin D (cholecalciferol 1500–2000 IU/day or alfacalcidol 0.5 μg/day). Due to severe back pain, a profound sense of maternal inadequacy, and significant psychological distress, three patients received additional bone-active therapies. Specifically, Case 1 (a *WNT1* pathogenic carrier) achieved pain relief and an improved femoral neck Z-score from -2.5 to -1.9 after 3 years of denosumab. Case 3 (another *WNT1* pathogenic variant carrier) attained complete pain resolution and a lumbar spine Z-score increase from -3.3 to -1.3 over 2 years via sequential therapy consisting of 8 months of teriparatide followed by three doses of denosumab. Similarly, Case 11 (an *LRP5* pathogenic variant carrier) underwent a 2-year regimen of romosozumab (12 months) and denosumab (three doses), resulting in complete pain relief and a lumbar spine Z-score increase from -3.5 to -2.4. Notably, Case 11 was the only patient with pre-conception densitometry data, having recorded a lumbar Z-score of -2.9 four years prior to pregnancy. Within the cohort, the eight patients managed solely with lactation cessation and supplementation demonstrated a significant lumbar spine Z-score improvement from -3.45 to -2.3 over the follow-up period (*P* = 0.012). When stratified by variant status, patients without DCVs exhibited a significant improvement from -3.0 to -2.0 (*P* = 0.043), while those with DCVs showed a non-significant change from -3.8 to -3.3 (*P* = 0.109). Furthermore, no statistically significant differences were observed between the conservative treatment and bone-active therapy groups regarding lumbar spine BMD Z-scores at baseline (-3.45 vs. -3.5, *P* = 0.538), at final follow-up (-2.3 vs. -1.85, *P* = 0.792), or in the magnitude of increase (0.7 vs. 1.55, *P* = 0.296). Overall, the entire cohort achieved complete back pain resolution and a statistically significant increase in median lumbar spine Z-scores (from -3.5 to -2.3, *P* = 0.005) at the median 25-month follow-up. Despite these improvements, skeletal recovery remained incomplete, with no individual achieving a Z-score above -1.0. During the follow-up period, no subsequent pregnancies or fracture recurrences were observed within the cohort.

**Figure 3:**
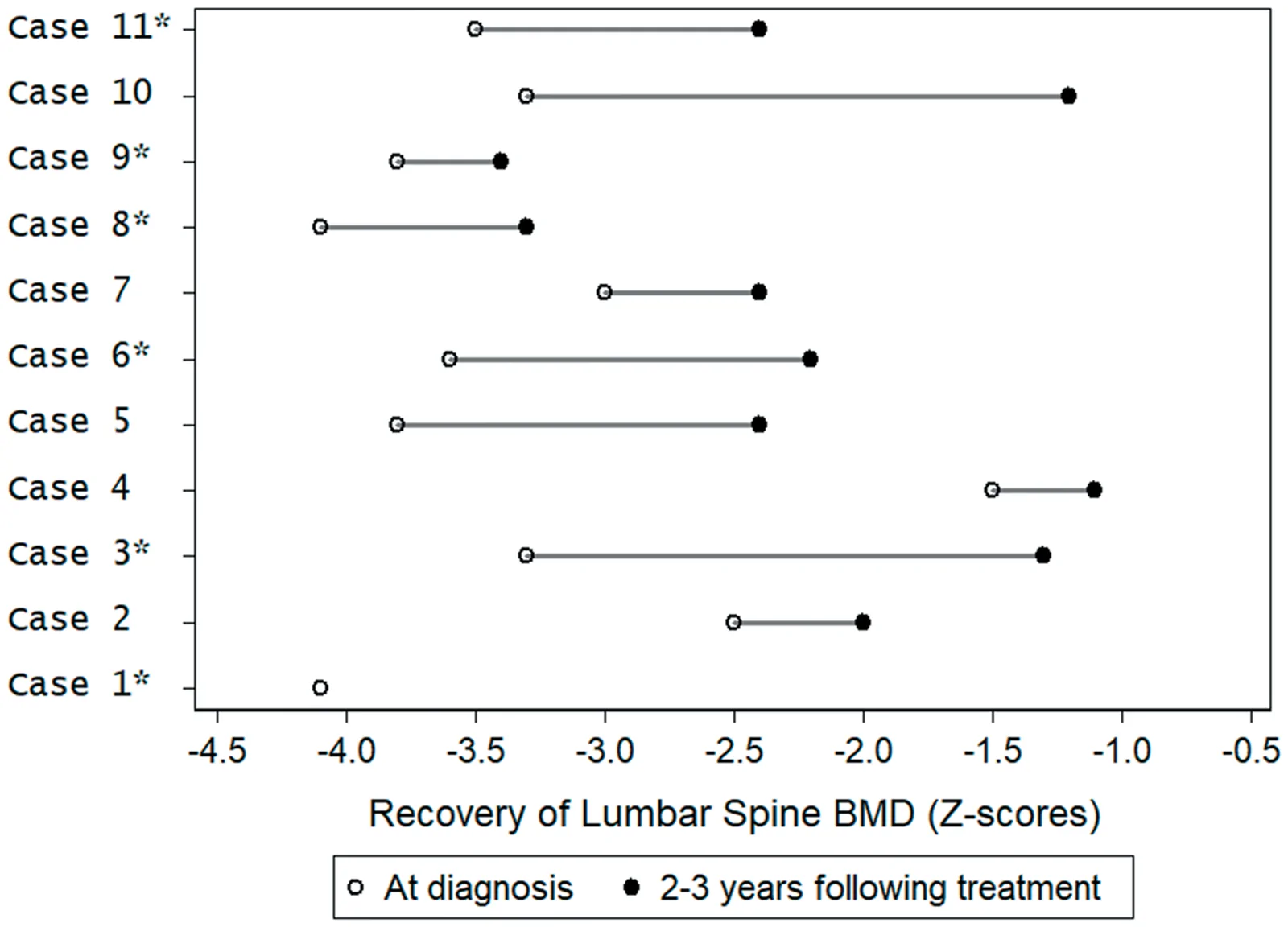
Clinical management and longitudinal skeletal outcomes of the PLO cohort. Asterisks (*) denote carriers of pathogenic DCVs. All individuals discontinued breastfeeding and initiated calcium and vitamin D supplementation upon diagnosis. Driven by severe back pain and secondary psychological distress, three patients (Cases 1, 3, and 11) received adjunctive bone-active therapies. At the 2-3-year follow-up, the entire cohort achieved complete back pain resolution and significantly improved bone mineral density (median lumbar spine Z-score increased from -3.5 to -2.3; *P* = 0.005), though individual Z-scores consistently remained below -1.0. Note: Lumbar spine densitometry was not assessed for Case 1; this individual exhibited an improved femoral neck Z-score from -2.5 to -1.9 at year three (data not shown).

## Discussion

PLO is a rare and heterogeneous disorder, with accumulating evidence implicating genetic susceptibility in its pathogenesis (7). Previous exome-sequencing studies have reported a high prevalence of skeletal disorder-associated variants in women with PLO, ranging from 30% in a North American cohort (9) and 50% in a German cohort (8) to 78.6% in a Chinese cohort (22), although these frequencies were derived using different analytical and classification frameworks. In our cohort, RVs were identified in 10 of 11 patients (91%), and six patients (55%) carried variants classified as DCVs under our predefined framework. Patients with DCVs exhibited significantly lower lumbar spine BMD, together with a trend toward both a younger age at PLO diagnosis and a higher proportion of multiple vertebral fractures than those without, suggesting a genetic contribution to disease severity. While the high variant frequency in our cohort suggests a significant genetic component in PLO, these findings warrant caution due to the small sample size. This observation creates an opportunity for pre-pregnancy genetic testing in women with existing fractures or confirmed family history of monogenic bone disorders, potentially shifting the focus from broad prediction to diagnostic clarification and personalized monitoring. In light of the absence of current guidelines for targeted screening in high-risk women, ensuring adequate calcium and vitamin D supplementation before and during pregnancy, and avoiding breastfeeding postpartum, could represent a preliminary preventative strategy for high-risk women with pathogenic variants pending further validation. Furthermore, our observations are consistent with previous exome-sequencing studies (8, 9, 22) showing that variants in the WNT/β-catenin signaling cascade predispose women to pregnancy- and lactation-associated bone loss. This finding also aligns with genome-wide association studies and gene-based analyses on BMD and osteoporosis in the Taiwanese population, which have highlighted that WNT-mediated signaling is one of the pivotal pathways in bone health (23).

The WNT1 protein, a central regulator of the WNT/β-catenin pathway, is essential for osteoblast differentiation and bone formation (24). Variants in the *WNT1* gene have been widely linked to early-onset osteoporosis and osteogenesis imperfecta (25–27). We identified a recurrent heterozygous variant, NM_005430.4(*WNT1*):​c.677C>T ​p.(Ser226Leu), previously shown to impair WNT/β-catenin signaling and to downregulate BMP-2 expression in preosteoblasts (28, 29), and a second missense variant, NM_005430.4(*WNT1*):​c.291C>A​(p.Phe97Leu), which is absent from the ClinVar database (January 2025 release) and requires functional characterization. *LRP5* has been linked to both primary osteoporosis and PLO (30–32). Previous reports have described heterozygous and compound heterozygous *LRP5* variants that contribute to the clinical manifestation of PLO (33, 34). Here we identified a rare nonsense variant, NM_002335.4(*LRP5*):c.350G>A p.(Trp117*), which was not reported in ClinVar at the time of annotation and is predicted to truncate the LRP5 protein within its first β-propeller domain and thereby to impair WNT co-receptor function. Additionally, one patient in our cohort carried a heterozygous variant in NM_002335.4(*LRP5*):​c.3608C>T p.(Ala1203Val) alongside a heterozygous variant in NM_000493.4(*COL10A1*):​c.1228G>C​ p.(Gly410Arg). These two missense variants are previously unreported and require further cellular functional verification. *COL10A1* encodes type X collagen, which is crucial for endochondral ossification, and has been linked to both metaphyseal chondrodysplasia and osteoporosis (35). The co-occurrence of these variants suggests a possible polygenic contribution to skeletal fragility in this population. We also identified rare variants in *CALCR* and *LTBP3* genes implicated in bone remodeling. *CALCR* encodes the calcitonin receptor regulating osteoclast-mediated bone resorption (36). The nonsense variant NM_001164737.3(*CALCR*):​c.520A>T​ p.(Arg174*) truncates the receptor immediately before the position at which the two human calcitonin receptor isoforms differ by a 16-amino-acid insertion within the first intracellular loop (37, 38), and would be expected to abolish receptor signaling. Of note, this variant was detected at a variant allele fraction of 0.29, and although it was confirmed on manual review of the read alignments, somatic mosaicism or allelic imbalance in the capture data cannot be excluded without an independent orthogonal assay. *LTBP3* gene encodes latent transforming growth factor-beta binding protein 3 (LTBP3), a critical regulator of transforming growth factor-beta (TGF-β) bioavailability. Pathogenic variants in this gene are associated with skeletal dysplasia and low BMD (39). Mechanistically, LTBP3 deficiency may decrease active TGF-β in bone and cartilage, potentially impairing osteoblast and osteoclast function. Further research is warranted to validate the NM_001130144.3(*LTBP3*):​c.1936G>A​ p.(Gly646Ser) variant identified here in the pathogenesis of osteoporosis and fragility fractures. During pregnancy, bone turnover markers (formation and resorption) are elevated with consistently high parathyroid hormone-related protein driving calcium mobilization from maternal bone (1). While the specific interplay in human gestation remains uncharacterized, animal models suggest that *CALCR* gene expression appears upregulated (2- to 3-fold) in both cortical and trabecular bones at the end of pregnancy and lactation compared to virgin controls, expressed specifically by osteoclasts (40), with the potential to restrict excessive bone resorption. In addition, studies in osteoporotic rats indicate that calcitonin receptor activation could upregulate WNT ligands (41), suggesting a potential synergistic mechanism for preserving skeletal integrity under physiological stress. However, direct evidence for such a CALCR-WNT/LRP5 interplay during human pregnancy is currently lacking and warrants further investigation. Furthermore, during lactation, animal models have demonstrated that osteoclast-mediated bone resorption (mainly in trabecular bone) and osteocytic osteolysis (in trabecular and cortical bones) mobilize maternal bone minerals (42–45), while hypothalamic cellular communication network factor 3 (CCN3) signaling drives compensatory bone formation (46). This delicate balance involves LTBP3 and COL10A1 during local matrix remodeling, incorporates CALCR signaling to restrict excessive resorption, and features CCN3 cross-talk with the WNT1/LRP5 signaling axis to stimulate osteoblastic recovery throughout the breastfeeding period. Together, these findings support that PLO is not just a “mechanical” result of pregnancy/lactation, but often has a pre-existing genetic susceptibility.

During lactation, the physiological mobilization of calcium occurs preferentially from trabecular bone (42–45), a pattern reflected in our cohort by significantly lower lumbar spine Z-scores compared to the hip. Consistent with previous reports (6), vertebral fractures were the predominant manifestation of PLO, often involving multiple thoracolumbar levels, though sacral alae and pubic bone fractures were also observed. Beyond physical disability, these fractures caused debilitating pain that severely impaired essential newborn care, such as lifting or bathing infants. This functional incapacity, compounded by the risk of fracture recurrence, often led to a profound sense of maternal inadequacy and significant psychological distress. Consequently, the management of PLO must integrate both skeletal stabilization and comprehensive psychosocial support for the mother.

In the absence of controlled trials, PLO screening and management remain empirical and lack established guidelines. A thorough investigation is necessary to identify potential contributing factors, such as underweight, advanced maternal age, nutritional deficiencies (calcium and vitamin D), sedentary lifestyle, smoking, chronic diseases, and previous fragility fractures (6, 47–50). Early lactation cessation and calcium/vitamin D supplementation constitute the mainstay of management (6, 47–49). Current evidence has not yet established a definitive role for bone formation or anti-resorptive agents in the management of PLO (51). In our cohort, patients managed solely with this conservative approach demonstrated significant lumbar BMD recovery over the follow-up period. While these preliminary observations suggest that conservative management could serve as a foundational strategy, our study lacked the statistical power to conclusively evaluate the therapeutic efficacy of adjunctive anabolic/anti-resorptive agents. Consequently, whether these therapies could alter the trajectory of BMD accrual, accelerate back pain resolution, or expedite the restoration of maternal function remains to be elucidated. Larger, multi-center datasets are required to fully clarify these therapeutic implications for PLO patients both with and without specific genetic risk variants.

Epidemiological studies indicate that lactation generally does not alter peak bone mass or long-term fracture risk because BMD typically recovers within 6–12 months after weaning (42). In our cohort, all 11 patients showed BMD improvement 25 months after weaning and treatment. However, none reached a Z-score above -1.0. This finding aligns with a recent systematic review (52) of 451 PLO patients (1988-2022). While that review noted significant overall BMD gains across treatments, evaluation of the compiled individual case profiles reveals that post-treatment lumbar Z-scores predominantly remained below -1.0. Although a Z-score above -2.0 formally defines bone density as “within the expected range for age” (10), a Z-score below -1.0 corresponds to the 16th percentile or lower of an age-matched population. This outcome may reflect low pre-pregnancy bone mass or incomplete skeletal recovery. While our cohort had no history of pre-pregnancy fractures, Case 11 was the sole patient with a documented baseline BMD. Her pre-pregnancy lumbar spine Z-score of -2.9 explicitly demonstrates that her bone density was compromised below the expected range for age before her pregnancy. Moreover, previous reports suggest that PLO may be a predictor of persistent osteopenia (53) and future fractures (54). Furthermore, high-resolution peripheral quantitative computed tomography (HR-pQCT) studies in lactating women have revealed persistent impairments in trabecular and cortical parameters. These findings suggest potential permanent alterations in bone microarchitecture (55, 56). While most women regain bone mass after weaning, our results and prior HR-pQCT data indicate that recovery may be incomplete in individuals with underlying genetic defects. This underscores the clinical value of pre-pregnancy BMD screening for identifying women at high risk for fragility fractures and highlights the necessity of long-term clinical monitoring.

### Study limitations

Our study has several limitations that warrant consideration. First, the sample size is relatively small (n = 11), which is a common constraint when investigating rare conditions such as PLO. The high detection rate of genetically relevant variants (91%) and pathogenic defects (55%) observed in our cohort suggests a potential genetic contribution to PLO, highlighting the need for larger studies. Second, no functional or segregation studies were performed. Variant classification relied on computational prediction and database evidence, and describes pathogenicity under the ACMG/AMP framework rather than a demonstrated contribution to the phenotype. Variants without prior PLO-specific functional or segregation evidence require functional validation and, where possible, family segregation analysis before their contribution to the PLO phenotype can be established. Genetic variants alone cannot establish causality in an observational cohort of this size. Third, our cohort was drawn entirely from an Asian population at a single tertiary medical center; consequently, these findings may not fully represent the genetic architecture or clinical progression of PLO in ethnically diverse populations. Fourth, because pre-pregnancy BMD data were unavailable for most participants, we could not definitively establish baseline skeletal health prior to the physiological stress of childbearing. Finally, while we observed significant BMD improvement over a 25-month follow-up period, longer-term longitudinal tracking is required to determine the lifetime fracture risk and the permanent trajectory of skeletal recovery in women harboring these specific genetic variants.

## Conclusion

Our study demonstrates that PLO is characterized by severe, multi-level vertebral fractures and a significant genetic burden. The high prevalence of genetic variants observed in this cohort suggests a potential genetic contribution to PLO, likely precipitated by the physiological stressors of pregnancy and lactation. Specific pathogenic variants indentified (*WNT1*, *LRP5*, *COL10A1*, *LTBP3*, *CALCR*) appear associated with phenotypic severity, offering preliminary insights into the connection between molecular discoveries and clinical presentation. The clinical hallmark of PLO is preferential trabecular bone loss, leading to a high frequency of vertebral fractures that cause debilitating pain and severely impair newborn care. Although timely lactation cessation and therapeutic interventions significantly improved lumbar BMD at a median 25-month longitudinal follow-up, skeletal recovery remains incomplete, with no patient achieving a Z-score above -1.0. Therefore, acute postpartum back pain warrants immediate investigation. Recognizing this underlying genetic burden warrants consideration of early intervention, long-term bone health monitoring, and future development of genotype-informed management strategies to potentially improve maternal outcomes.

## Data Availability

The original contributions presented in the study are included in the article/**Supplementary Material**. Further inquiries can be directed to the corresponding authors.
