# Supplementary material for: High genetic predisposition and vertebral fracture burden in pregnancy and lactation-associated osteoporosis: clinical insights from a Taiwanese cohort
Source: Front Endocrinol (Lausanne). 2026 Oct 6;17:1930247. doi: 10.3389/fendo.2026.1930247 (PMC13680257; doi:10.3389/fendo.2026.1930247)
Supplement: Supplementary file 1 [file DataSheet1.pdf]

Supplementary Materials

Supplementary Table 1. List of genes included in the 398-gene skeletal disease virtual panel for exome sequencing annotation

ABCC6, ACAN, ACP5, ACVR1, ADAMTS10, ADAMTS17, AFF4, AGA, AGPS, AIFM1, ALPI, ALPL, ALS2, AMER1, ANKH, ANO5, AP2S1, ARCN1, ARSB, ARSE, ASCC1, ASPM, ATP6V0A2, ATP7A, ATR, ATRIP, B3GALT6, B3GAT3, B4GALT7, BGN, BMP1, BMP2, BMPER, BMPR1B, C2CD3, CA2, CALCR, CANT1, CASR, CCDC8, CCN3, CDC45, CDC6, CDK5RAP2, CDKN1C, CDT1, CENPJ, CEP120, CEP135, CEP152, CEP63, CEP97, CFAP410, CHST14, CHST3, CHUK, CLCN5, CLCN7, COG1, COL10A1, COL11A1, COL11A2, COL1A1, COL1A2, COL27A1, COL2A1, COL3A1, COL5A1, COL5A2, COL9A1, COL9A2, COL9A3, COMP, CREB3L1, CRIPT, CRTAP, CSF1R, CSGALNACT1, CSPP1, CTNS, CTSA, CTSK, CUL7, CWC27, CYP24A1, CYP27B1, CYP2R1, DDR2, DDRGK1, DHCR24, DIP2C, DLL1, DLL3, DLX3, DMP1, DMRT2, DNA2, DNMT3A, DONSON, DSPP, DVL1, DVL3, DYM, DYNC2H1, DYNC2LI1, EBP, EIF2AK3, ENPP1, ESCO2, ESR1, EVC, EVC2, EXOC6B, EXOSC2, EXT1, EXT2, EXTL3, FAH, FAM111A, FAM20C, FAM46A, FAR1, FAT4, FBN1, FERMT3, FGF23, FGF9, FGFR1, FGFR2, FGFR3, FIG4, FKBP10, FLNA, FLNB, FN1, FTO, FUCA1, FZD2, GALNS, GALNT3, GDF5, GDF6, GHR, GHRHR, GHSR, GJA1, GLB1, GMNN, GNA11, GNAS, GNE, GNPAT, GNPTAB, GNPTG, GNS, GORAB, GPAA1, GPC6, GPX4, GSC, GUSB, GZF1, HES7, HGSNAT, HPGD, HRAS, HSPG2, HYAL1, IARS2, ICK, IDS, IDUA, IFIH1, IFITM5, IFT122, IFT140, IFT172, IFT43, IFT52, IFT57, IFT74, IFT80, IFT81, IGF1, IGF2, IHH, IL6, IMPAD1, INPPL1, INTU, JAG1, KAT6B, KDELR2, KIAA0586, KIAA0753, KIF22, KISS1R, KL, KMT2A, KRAS, LARP7, LBR, LEMD3, LFNG, LIFR, LIG4, LMNA, LMX1B, LONP1, LOXL3, LRP4, LRP5, LRRK1, LTBP2, LTBP3, MAFB, MAN2B1, MANBA, MAP3K7, MATN3, MBTPS1, MBTPS2, MCM5, MCPH1, MEOX1, MEPE, MESD, MESDC2, MESP2, MGP, MMP13, MMP14, MMP2, MMP9, MNX1, MSX2, MTAP, MYH3, MYO18B, NAGLU, NANS, NBAS, NEK1, NEU1, NHERF1, NIN, NKX3-2, NOG, NOTCH2, NPPC, NPR2, NPR3, NRAS, NSDHL, NSMCE2, NTRK1, NXN, OBSL1, OCRL, ORC1, ORC4, ORC6, OSTM1, P3H1, P4HB, PAM16, PAPSS2, PCGF2, PCNT, PCYT1A, PDE4D, PDLIM4, PEX5, PEX7, PGM3, PHEX, PIK3C2A, PISD, PKDCC, PLEKHM1, PLK4, PLOD2, PLOD3, PLS3, POC1A, POLR1A, POP1, POR, PPIB, PPP3CA, PRKAR1A, PTDSS1, PTH1R, PTHLH, PTPN11, PYCR1, RAB33B, RBBP8, RECQL4, RIL, RIPPLY2, RMRP, RNU4ATAC, ROR2, RSPO2, RSPRY1, RTTN, RUNX2, SC5D, SEC24D, SERPINF1, SERPINH1, SETBP1, SFRP4, SGMS2, SGSH, SH3PXD2B, SLC17A5, SLC26A2, SLC29A3, SLC2A2, SLC34A1, SLC34A3, SLC35D1, SLC39A13, SLC5A6, SLC9A3R1, SLCO2A1, SLCO5A1, SMAD4, SMARCAL1, SMS, SNRPB, SNX10, SOST, SOX9, SP7, SPARC, SQSTM1, SRCAP, SUCO, SULF1, SUMF1, TAB2, TAPT1, TBCE, TBX15, TBX3, TBX5, TBX6, TBXAS1, TCIRG1, TCTEX1D2, TCTN3, TENT5A, TGFB1, TMEM165, TMEM38B, TNFRSF11A, TNFRSF11B, TNFSF11, TONSL, TRAF3IP1, TRAF6, TRAIP, TRAPPC2, TREM2, TRIM37, TRIP11, TRIP4, TRMT10A, TRPS1, TRPV4, TRPV6, TTC21B, TUBGCP4, TUBGCP6, TYROBP, UBE3B, VAC14, VDR, VPS33A, WDR19, WDR34, WDR35, WDR4, WDR60, WISP3, WNT1, WNT3, WNT3A, WNT5A, XRCC4, XYLT1, XYLT2, ZBTB20, ZMPSTE24, ZNF687

Supplementary Figure 1

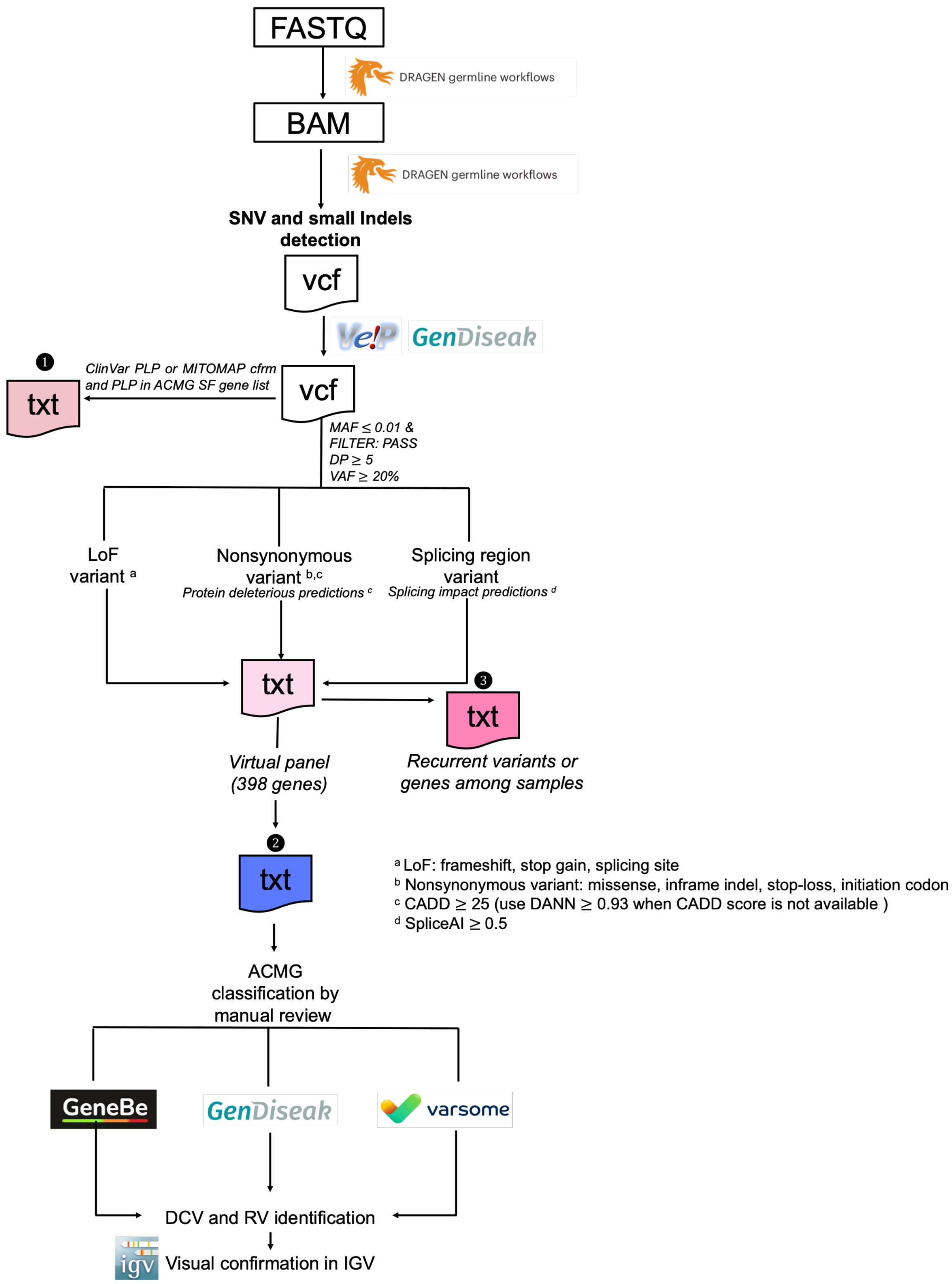

Supplementary Figure 1. **Whole-exome sequencing analysis pipeline and variant filtering workflow.** Raw sequencing reads (FASTQ) were aligned and processed using the DRAGEN germline workflows to generate BAM files, from which single-nucleotide variants (SNVs) and small insertions/deletions were called (VCF). Called variants were annotated with the Variant Effect Predictor (VEP) with Gendiseak

platform. Variants entered the primary prioritization pathway only if they carried a FILTER status of "PASS", a minor allele frequency (MAF) ≤ 0.01 in gnomAD, a read depth (DP) ≥ 5, and a variant allele fraction (VAF) ≥ 20%. Retained variants were triaged into three classes: loss-of-function (LoF) variants (a); nonsynonymous variants (b) predicted to be protein-damaging (c); and splicing-region variants with a predicted splicing impact (d). The pooled candidate set was then restricted to the 398-gene skeletal disease virtual panel (Supplementary Table 1) (❷) and was screened in parallel for variants or genes recurring across samples (❸). Panel-restricted candidates were classified according to the five-tier ACMG/AMP system by manual review, cross-checked against ClinVar, VarSome, GeneBe and Gendiseak, to define relevant variants (RVs; ACMG/AMP classes II–V) and disease-causing variants (DCVs; classes IV–V). All variants retained after classification were confirmed by manual inspection of read alignments in the Integrative Genomics Viewer (IGV). In silico predictions were used only as supporting evidence within the ACMG/AMP framework and were never used in isolation to assign pathogenicity.

A second, parallel pathway (❶) reviewed variants already reported as pathogenic or likely pathogenic (PLP) in ClinVar, confirmed (cfrm) in MITOMAP, or PLP in a gene on the ACMG secondary findings (SF) list, independently of the allele-frequency filter. This pathway served as a supplementary check of the annotation pipeline and did not form part of the primary analysis; no variant relevant to PLO and no reportable secondary finding was identified through it, and its output is not included in the variant counts reported in the Results.

Figure footnotes: [a]LoF: frameshift, stop-gain, splicing site. [b]Nonsynonymous variant: missense, in-frame indel, stop-loss, initiation codon. [c]CADD phred ≥ 25 (DANN ≥ 0.93 used where a CADD score was unavailable). [d]SpliceAI ≥ 0.5.

Abbreviations: ACMG/AMP, American College of Medical Genetics and Genomics/Association for Molecular Pathology; BAM, Binary Alignment Map; CADD, Combined Annotation Dependent Depletion; cfrm, confirmed; DANN, Deleterious Annotation of genetic variants using Neural Networks; DCV, disease-causing variant; DP, read depth; IGV, Integrative Genomics Viewer; LoF, loss of function; MAF, minor allele frequency; PLP, pathogenic or likely pathogenic; RV, relevant variant; SF, secondary findings; SNV, single-nucleotide variant; VAF, variant allele fraction; VCF, Variant Call Format; VEP, Variant Effect Predictor.
